# Supplementary figures and images for: Diversity and structure of the root-associated bacterial microbiomes of four mangrove tree species, revealed by high-throughput sequencing
Source: PeerJ. 2023 Oct 4;11:e16156. doi: 10.7717/peerj.16156 (PMC10559887; doi:10.7717/peerj.16156)

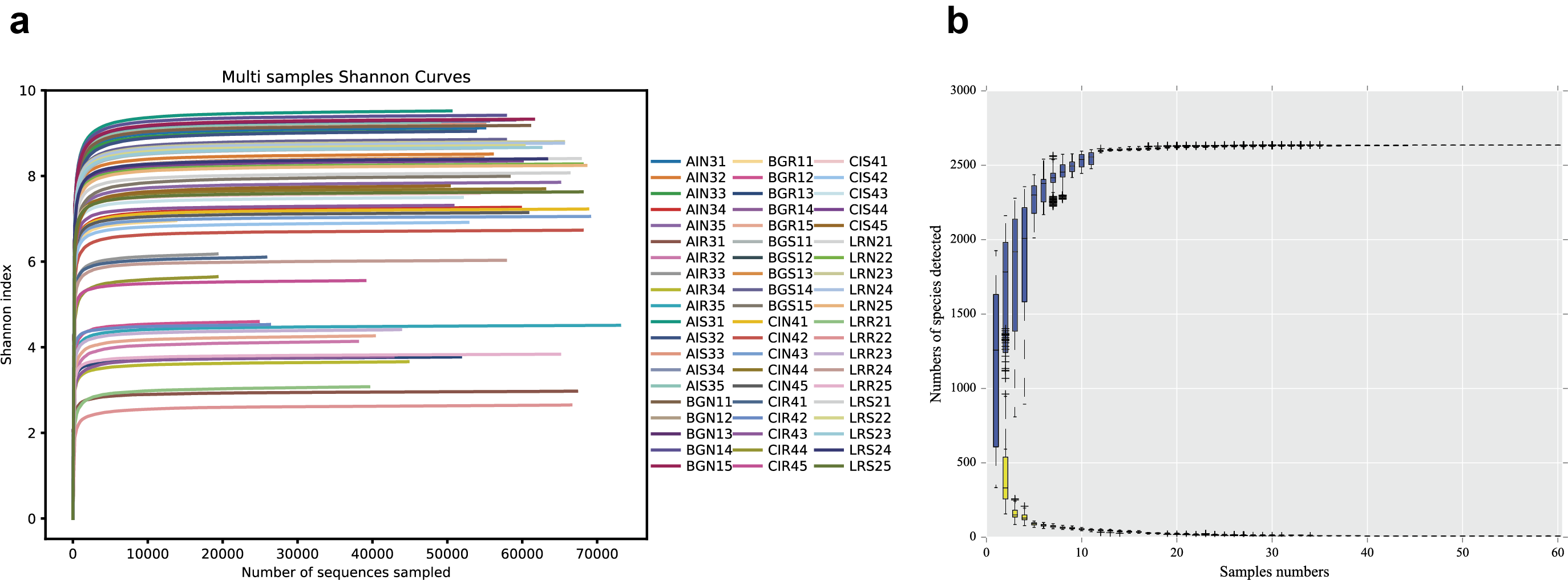

Supplement: Supplemental Information 1 [file peerj-11-16156-s001.png]

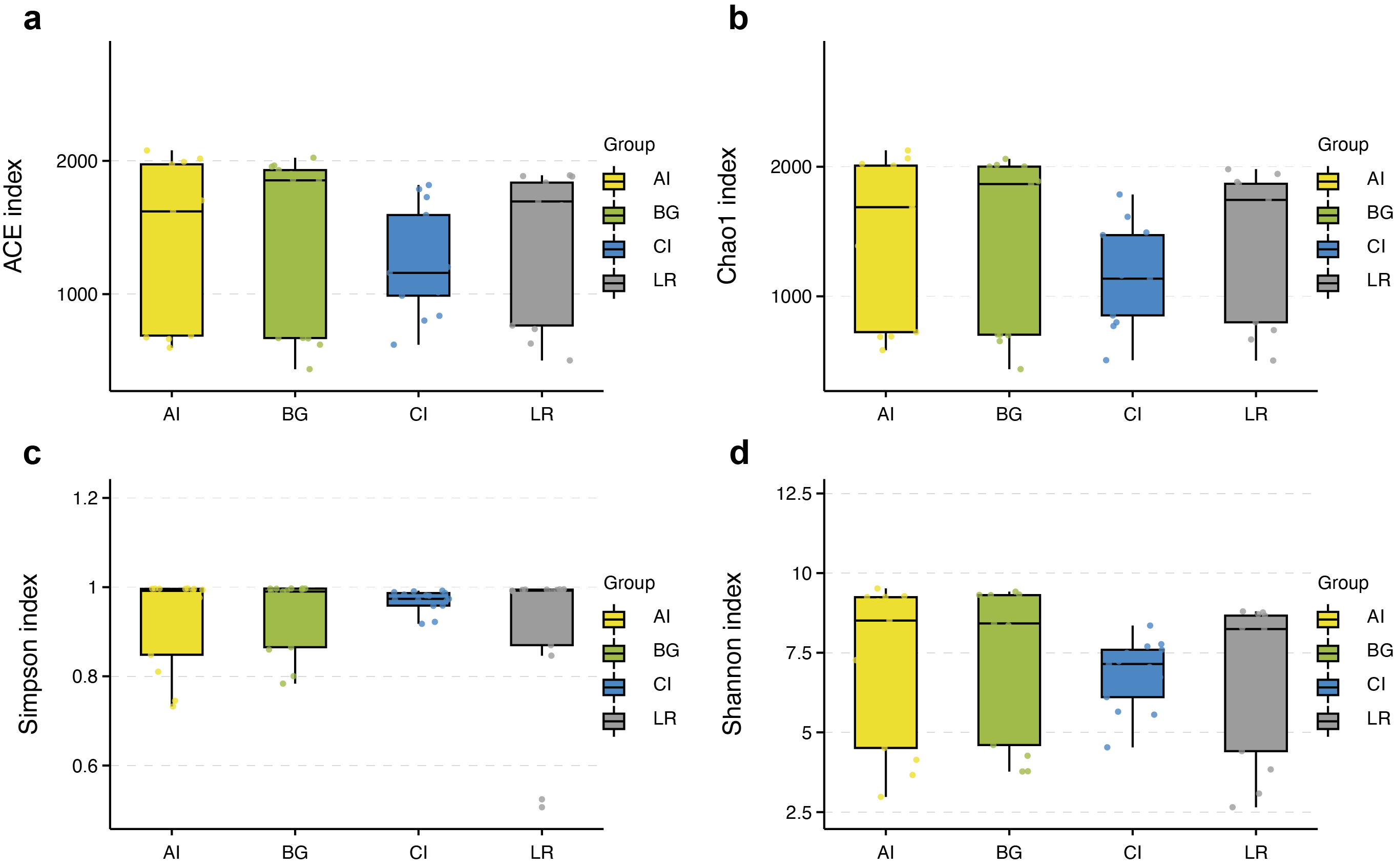

Supplement: Supplemental Information 2 — (A–D) depict ACE, Chao1, Simpson, and Shannon index of four different mangrove species in all samples, respectively. The horizontal bars within boxes represent median. The tops and bottoms of boxes represent the 75th and 25th quartiles, respectively. The upper and lower whiskers extend 1.5 × the interquartile range from the upper edge and lower edge of the box, respectively. All samples are plotted as individual points. [file peerj-11-16156-s002.png]

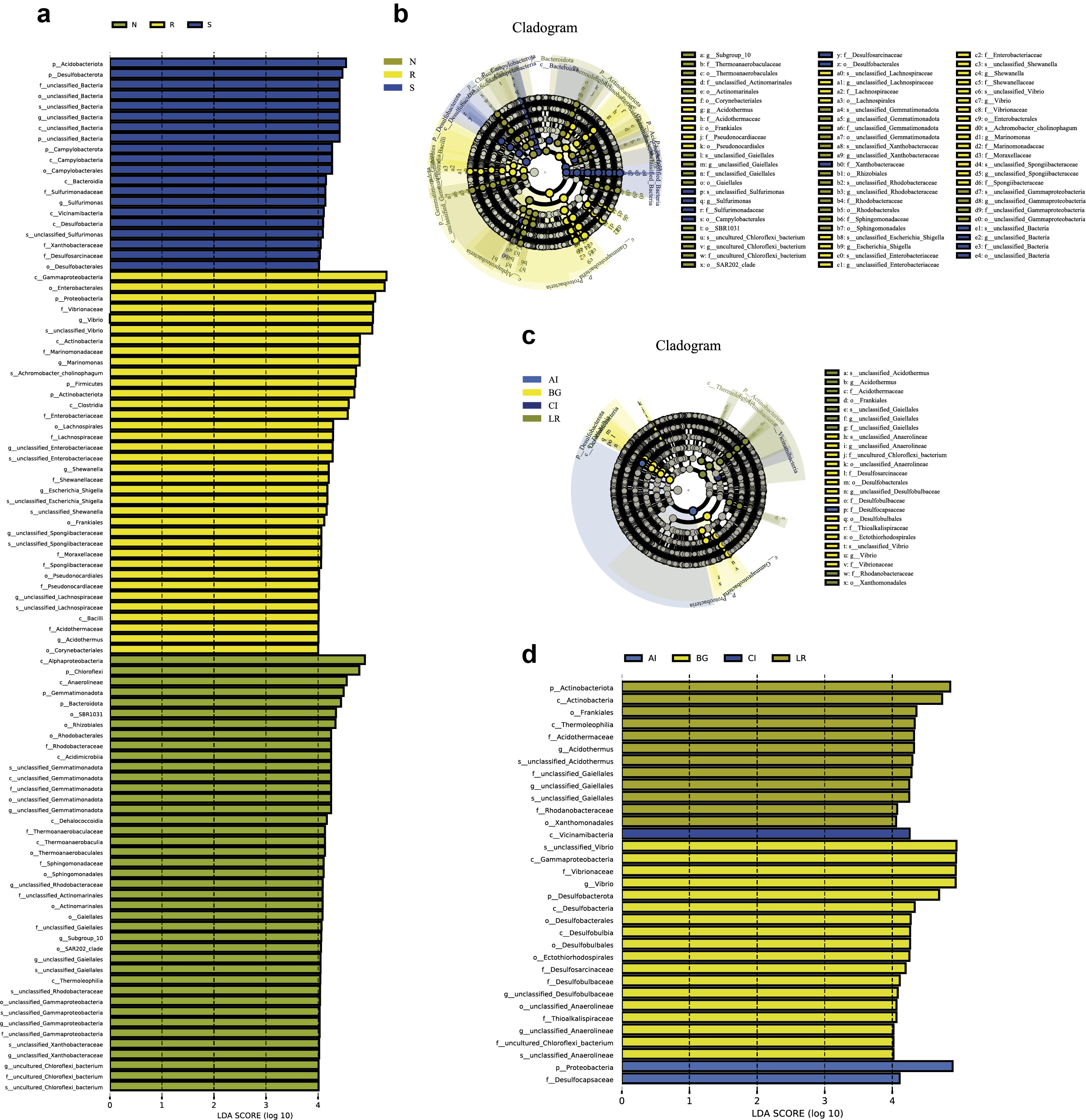

Supplement: Supplemental Information 3 — (A) LEfSe cladogram indicating the phylogenetic distribution of taxon associated with the three compartments or the four mangrove species (logarithmic LDA score ≥ 4.0 and P ≤ 0.05). Circles from center to outward layers represent taxonomic level from phylum to species. Differences are represented in the color of the most abundant class. The diameter of each circle is proportional to the abundance of the taxon. The node size represents the difference in relative abundance; (B) Histogram of differentially abundant features between groups (logarithmic LDA score ≥ 4.0 and P ≤ 0.05). A longer bar indicates a more significant difference. The bars were coloured according to the group with highest abundance of corresponding feature. p, phylum; c, class; o, order; f, family; g, genus; s, species. [file peerj-11-16156-s003.png]
