# Supplementary material for: Diversity and structure of the root-associated bacterial microbiomes of four mangrove tree species, revealed by high-throughput sequencing
Source: PeerJ. 2023 Oct 4;11:e16156. doi: 10.7717/peerj.16156 (PMC10559887; doi:10.7717/peerj.16156)
Supplement: Supplemental Information 4 — OTU_Num refers to the number of operational taxonomic units (OTUs); Seqs_Num refers to the number of reads corresponding to assigned OTUs; N, S, and R refer to non-rhizosphere soil, rhizosphere soil and endosphere, respectively. [file peerj-11-16156-s004.doc]

| Sample ID | compartment | Raw Reads | Clean Reads | Effective Reads | AvgLen (bp) | GC(%) | Q20(%) | Q30(%) | Effective(%) | OTU_Num | Seqs_Num |
| --- | --- | --- | --- | --- | --- | --- | --- | --- | --- | --- | --- |
| AIN31 | N | 79851 | 79571 | 78499 | 420 | 56.43 | 99.2 | 96.56 | 98.31 | 1689 | 55064 |
| AIN32 | N | 80204 | 79870 | 78469 | 419 | 56.51 | 99.19 | 96.56 | 97.84 | 1391 | 56165 |
| AIN33 | N | 79887 | 79615 | 78483 | 420 | 56.37 | 99.21 | 96.61 | 98.24 | 1570 | 51985 |
| AIN34 | N | 80022 | 79713 | 76830 | 415 | 56.72 | 99.24 | 96.73 | 96.01 | 1145 | 59930 |
| AIN35 | N | 80275 | 80004 | 78601 | 414 | 57 | 99.24 | 96.72 | 97.91 | 1299 | 65121 |
| AIR31 | R | 82925 | 82623 | 76588 | 425 | 53.65 | 99.13 | 96.41 | 92.36 | 495 | 67401 |
| AIR32 | R | 54460 | 54344 | 47933 | 424 | 53.81 | 99.17 | 96.54 | 88.02 | 573 | 38119 |
| AIR33 | R | 34771 | 34671 | 26242 | 423 | 54.43 | 99.19 | 96.6 | 75.47 | 574 | 19360 |
| AIR34 | R | 56742 | 56610 | 50721 | 427 | 54.46 | 99.22 | 96.68 | 89.39 | 457 | 44954 |
| AIR35 | R | 89793 | 89585 | 86231 | 422 | 56.06 | 99.19 | 96.69 | 96.03 | 649 | 73135 |
| AIS31 | S | 79993 | 79707 | 78529 | 422 | 56 | 99.2 | 96.56 | 98.17 | 1926 | 50508 |
| AIS32 | S | 79768 | 79471 | 77898 | 421 | 55.13 | 99.19 | 96.52 | 97.66 | 1870 | 53761 |
| AIS33 | S | 80202 | 79893 | 78849 | 421 | 56.21 | 99.16 | 96.46 | 98.31 | 1798 | 55082 |
| AIS34 | S | 80648 | 80360 | 78717 | 420 | 55.73 | 99.2 | 96.59 | 97.61 | 1780 | 55643 |
| AIS35 | S | 80004 | 79724 | 78134 | 422 | 55.6 | 99.16 | 96.47 | 97.66 | 1719 | 49216 |
| BGN11 | N | 79946 | 79607 | 78388 | 420 | 55.6 | 99.17 | 96.49 | 98.05 | 1751 | 61025 |
| BGN12 | N | 79579 | 79298 | 78043 | 420 | 56.31 | 99.19 | 96.56 | 98.07 | 1841 | 60019 |
| BGN13 | N | 79925 | 79593 | 78545 | 420 | 55.34 | 99.17 | 96.49 | 98.27 | 1791 | 59187 |
| BGN14 | N | 80022 | 79722 | 78643 | 421 | 55.71 | 99.16 | 96.45 | 98.28 | 1836 | 57991 |
| BGN15 | N | 79862 | 79555 | 78314 | 421 | 55.92 | 99.19 | 96.54 | 98.06 | 1791 | 61591 |
| BGR11 | R | 30415 | 30289 | 28244 | 418 | 56.36 | 99.21 | 96.62 | 92.86 | 572 | 13975 |
| BGR12 | R | 30328 | 30222 | 29512 | 426 | 53.86 | 99.15 | 96.38 | 97.31 | 369 | 24986 |
| BGR13 | R | 58367 | 58205 | 57438 | 428 | 52.51 | 99.15 | 96.39 | 98.41 | 438 | 51852 |
| BGR14 | R | 53843 | 53706 | 51677 | 427 | 53.53 | 99.15 | 96.43 | 95.98 | 552 | 42811 |
| BGR15 | R | 50594 | 50472 | 49463 | 425 | 53.73 | 99.18 | 96.5 | 97.76 | 563 | 40458 |
| BGS11 | S | 79860 | 79538 | 78159 | 419 | 55.28 | 99.17 | 96.49 | 97.87 | 1582 | 54648 |
| BGS12 | S | 80036 | 79711 | 76082 | 420 | 55.38 | 99.15 | 96.42 | 95.06 | 1657 | 57739 |
| BGS13 | S | 80201 | 79898 | 78405 | 419 | 55.16 | 99.18 | 96.53 | 97.76 | 1550 | 54921 |
| BGS14 | S | 79868 | 79578 | 78520 | 420 | 55.42 | 99.2 | 96.59 | 98.31 | 1669 | 57965 |
| BGS15 | S | 79986 | 79644 | 78322 | 419 | 55.13 | 99.2 | 96.6 | 97.92 | 1599 | 58394 |
| CIN41 | N | 80132 | 79848 | 79005 | 415 | 58.33 | 99.24 | 96.73 | 98.59 | 1137 | 68793 |
| CIN42 | N | 80103 | 79778 | 78750 | 421 | 58.61 | 99.16 | 96.48 | 98.31 | 956 | 68026 |
| CIN43 | N | 79769 | 79482 | 78362 | 417 | 58.95 | 99.2 | 96.58 | 98.24 | 864 | 69226 |
| CIN44 | N | 80077 | 79775 | 78763 | 418 | 58.46 | 99.21 | 96.63 | 98.36 | 1039 | 63135 |
| CIN45 | N | 79665 | 79354 | 77043 | 416 | 57.62 | 99.21 | 96.64 | 96.71 | 982 | 60971 |
| CIR41 | R | 39413 | 39296 | 37304 | 417 | 56.69 | 99.27 | 96.94 | 94.65 | 561 | 25847 |
| CIR42 | R | 33490 | 33361 | 31276 | 412 | 53.31 | 99.19 | 96.55 | 93.39 | 333 | 26319 |
| CIR43 | R | 79476 | 79298 | 76091 | 419 | 57.1 | 99.24 | 96.82 | 95.74 | 731 | 50847 |
| CIR44 | R | 28508 | 28430 | 27464 | 422 | 55.95 | 99.21 | 96.74 | 96.34 | 624 | 19403 |
| CIR45 | R | 53566 | 53421 | 49306 | 423 | 55.78 | 99.22 | 96.73 | 92.05 | 538 | 39138 |
| CIS41 | S | 80442 | 80148 | 79447 | 418 | 57.71 | 99.22 | 96.67 | 98.76 | 1014 | 54275 |
| CIS42 | S | 79596 | 79262 | 78378 | 418 | 56.44 | 99.19 | 96.55 | 98.47 | 1024 | 52772 |
| CIS43 | S | 80345 | 80086 | 78865 | 416 | 57.18 | 99.23 | 96.71 | 98.16 | 927 | 52008 |
| CIS44 | S | 80150 | 79878 | 78675 | 417 | 57.15 | 99.23 | 96.71 | 98.16 | 1469 | 60114 |
| CIS45 | S | 80333 | 80040 | 76659 | 419 | 55.73 | 99.19 | 96.54 | 95.43 | 1382 | 50269 |
| LRN21 | N | 79740 | 79442 | 77800 | 420 | 56.7 | 99.21 | 96.59 | 97.57 | 1449 | 67844 |
| LRN22 | N | 80039 | 79738 | 78508 | 419 | 57.12 | 99.21 | 96.62 | 98.09 | 1446 | 68182 |
| LRN23 | N | 79760 | 79483 | 78015 | 419 | 56.15 | 99.2 | 96.56 | 97.81 | 1629 | 65709 |
| LRN24 | N | 80080 | 79762 | 78230 | 420 | 56.03 | 99.19 | 96.55 | 97.69 | 1640 | 65614 |
| LRN25 | N | 80227 | 79947 | 78464 | 419 | 56.79 | 99.23 | 96.65 | 97.8 | 1476 | 68601 |
| LRR21 | R | 51217 | 51100 | 49040 | 425 | 54.26 | 99.22 | 96.71 | 95.75 | 532 | 39685 |
| LRR22 | R | 77877 | 77721 | 74040 | 427 | 53.64 | 99.23 | 96.74 | 95.07 | 632 | 66516 |
| LRR23 | R | 52801 | 52683 | 50497 | 422 | 55.84 | 99.25 | 96.82 | 95.64 | 499 | 43751 |
| LRR24 | R | 83553 | 83354 | 73680 | 414 | 57.21 | 99.26 | 96.96 | 88.18 | 619 | 57989 |
| LRR25 | R | 71834 | 71683 | 70157 | 419 | 58.39 | 99.26 | 96.95 | 97.67 | 416 | 65182 |
| LRS21 | S | 79999 | 79722 | 78105 | 419 | 57.66 | 99.24 | 96.7 | 97.63 | 1391 | 66433 |
| LRS22 | S | 79953 | 79654 | 78352 | 421 | 55.79 | 99.19 | 96.53 | 98 | 1668 | 60466 |
| LRS23 | S | 79874 | 79559 | 78004 | 421 | 55.7 | 99.18 | 96.52 | 97.66 | 1607 | 62541 |
| LRS24 | S | 80014 | 79727 | 78428 | 418 | 56.59 | 99.25 | 96.75 | 98.02 | 1424 | 63380 |
| LRS25 | S | 80010 | 79697 | 77474 | 421 | 57.17 | 99.24 | 96.7 | 96.83 | 1217 | 68210 |
| Total | \ | \ | \ | \ | \ | \ | \ | \ | \ | 2636 | 3244252 |
