# Supplementary material for: Diversity and structure of the root-associated bacterial microbiomes of four mangrove tree species, revealed by high-throughput sequencing
Source: PeerJ. 2023 Oct 4;11:e16156. doi: 10.7717/peerj.16156 (PMC10559887; doi:10.7717/peerj.16156)
Supplement: Supplemental Information 5 — AI, BG, CI, and LR refer to A. ilicifolius, B. gymnorrhiza, C. inerme and L. racemosa, respectively; N, S, and R refer to non-rhizosphere soil, rhizosphere soil and endosphere, respectively. [file peerj-11-16156-s005.doc]

| Sample ID | Feature | ACE | Chao1 | Simpson | Shannon | Coverage |
| --- | --- | --- | --- | --- | --- | --- |
| AI |  |  |  |  |  |  |
| R | 1094 | 1231.107 | 1267.4688 | 0.9414 | 5.4082 | 0.9992 |
| S | 2273 | 2392.7657 | 2405.8306 | 0.9975 | 9.6795 | 0.9993 |
| N | 2091 | 2206.2105 | 2233.9106 | 0.9946 | 8.9409 | 0.9993 |
| BG |  |  |  |  |  |  |
| R | 975 | 1121.2672 | 1146.3011 | 0.8815 | 4.8868 | 0.999 |
| S | 2128 | 2288.8487 | 2341.0652 | 0.9939 | 8.7493 | 0.9991 |
| N | 2205 | 2353.4254 | 2398 | 0.9973 | 9.532 | 0.9994 |
| CI |  |  |  |  |  |  |
| R | 1136 | 1397.2977 | 1377.0084 | 0.9798 | 7.3539 | 0.9985 |
| S | 2034 | 2202.8389 | 2212.9032 | 0.9949 | 8.868 | 0.9991 |
| N | 1693 | 1835.7767 | 1869.75 | 0.9914 | 8.1146 | 0.9994 |
| LR |  |  |  |  |  |  |
| R | 1041 | 1213.58 | 1251.45 | 0.8672 | 5.0661 | 0.9993 |
| S | 2174 | 2346.7548 | 2360.0353 | 0.9951 | 8.8198 | 0.9992 |
| N | 2097 | 2254.2597 | 2262 | 0.9945 | 8.7319 | 0.9993 |
